# Supplementary material for: Differential negative dominance by KCNA2 variants associated with global developmental delay suggests KCNA2 haploinsufficiency in humans
Source: J Physiol. 2026 Mar 31;604(8):3413–30. doi: 10.1113/JP290728 (PMC13082188; doi:10.1113/JP290728)
Supplement: Supplementary file 2 — Supporting Information [file TJP-604-3413-s002.pdf]

Online appendix for

Differential negative dominance by two *KCNA2* variants associated with global developmental delay suggests *KCNA2* haploinsufficiency in humans

Pei Xin Boon, Amaia Jauregi-Miguel, Sümeyye Süheda Yasarbas, Serena Pozzi,
Urban Karlsson, Ammar Husami, Charmaine Ko, Amelle Shillington and Antonios Pantazis

This appendix includes extended clinical observations for the p.G318D patient.

APPENDIX

Clinical Summary:

This individual is a 10-year-old female with a known 17p12 microduplication involving the *PMP22* gene, consistent with Charcot-Marie-Tooth disease type 1A (CMT1A). Her additional medical history includes obstructive sleep apnea, duplicated left renal collecting system, laryngeal cleft, febrile seizures, and cortical visual impairment.

Developmental and Neurobehavioral Profile:

She attends an autism support classroom but has not received a formal autism spectrum disorder (ASD) diagnosis despite multiple evaluations. Parents and school personnel report social immaturity, distractibility, and sensory sensitivities, such as increased headphone use for sound modulation. Mother notes concern that patient's sociable and friendly demeanor may obscure underlying autistic features, particularly given her gender and mild presentation. Occupational therapy staff have noted attention difficulties and possible hearing-related inattention, though formal audiologic evaluation was normal.

Neurological and Musculoskeletal Findings:

Progressive gait instability and foot drop have been ongoing concerns, prompting reevaluation by the neuromuscular multidisciplinary team. Parents continued to question whether an additional diagnosis beyond CMT1A may contribute to her phenotype, given the degree of weakness and coordination abnormalities. She also exhibits muscle twitching during sleep, previously interpreted as periodic limb movement disorder (PLMD) on polysomnography, with possible neuromyotonic features, for this she is prescribed gabapentin, clonidine, melatonin and ferritin There have been no progressive seizure concerns since her prior febrile seizure history, though intermittent staring episodes persist. She was weaned off of all early anti-epileptic medication. Updated serial casting and new orthotic bracing have improved ambulation. She is on CoQ10 supplements for muscle weakness.

Growth and General Health:

The patient has demonstrated normal linear growth and healthy weight gain. Nutritional interventions, including additional snacks at school, have been beneficial. There have been no intercurrent illnesses and overall clinical stability has been maintained. She was found to have a redundant colon on gastrointestinal imaging and is managed for constipation with laxative interventions.

Previous Assessment and Genetic Investigation:

Given the multisystem phenotype not fully explained by her *PMP22* duplication, research-based genomic sequencing was pursued to evaluate for a second genetic etiology. Results identified a *de novo* variant of interest in *KCNA2* c.953G>A(p.Gly318Asp) (Fig.S1).

EEG description:

HISTORY: AL is a 6 y.o. 8 m.o. female who had a continuous video EEG at the request of Dr. Wei Kun Liu for spells versus seizures. For detailed history see admission history. This EEG was done from 8/25/2022 10:59 to 8/26/2022 06:12.

TECHNICAL: This 21-channel EEG was performed with a 32-channel digital EEG machine with electrodes placed according to the estimated international 10-20 system of placement. The study was done with time-locked video. Another channel was used for EKG. Electrodes T1 and T2 were also used. The data were stored digitally and reviewed in reformatted montages for optimal display. Electrodes were placed and removed in person by an EEG technologist. The criteria for unmonitored EEG review by an individual qualified in seizure recognition were met. The EEG and video were reviewed by the physician following conclusion of the study.

BACKGROUND: In the awake state the posterior dominant rhythm was a well-developed, rhythmic, moderate voltage 9 Hz activity that attenuated normally on eye opening. During the recording the patient entered N1 sleep and the background rhythm waxed and waned. During N2 sleep, well-developed sleep spindles were seen in the central head regions. V-waves and K-complexes were seen. Sleep N3 was recorded and was characterized by diffuse high amplitude delta activity. Photic stimulation was performed in the frequency range 3-30 Hz and produced a symmetric driving response in the posterior head regions. Hyperventilation was performed for 3 minutes and produced intermittent diffuse rhythmic slowing of the background activity. The patient's heart rate and rhythm appeared regular during the recording.

INTERICTAL: No focal slowing was seen. During REM sleep, rare bursts of high amplitude 4-5 Hz polymorphic delta with admixed spikes were seen diffusely bilaterally.

ICTAL: No electrographic or electroclinical seizures were noted.

OTHER EVENT(S): No other events were reported.

IMPRESSION: This was an abnormal EEG for age. Wakefulness, N1 sleep, N2 sleep, and N3 sleep were recorded. Rare burst of high amplitude 4-5 Hz polymorphic delta with admixed spikes were seen diffusely bilaterally, this is potentially epileptiform.

Figure S1

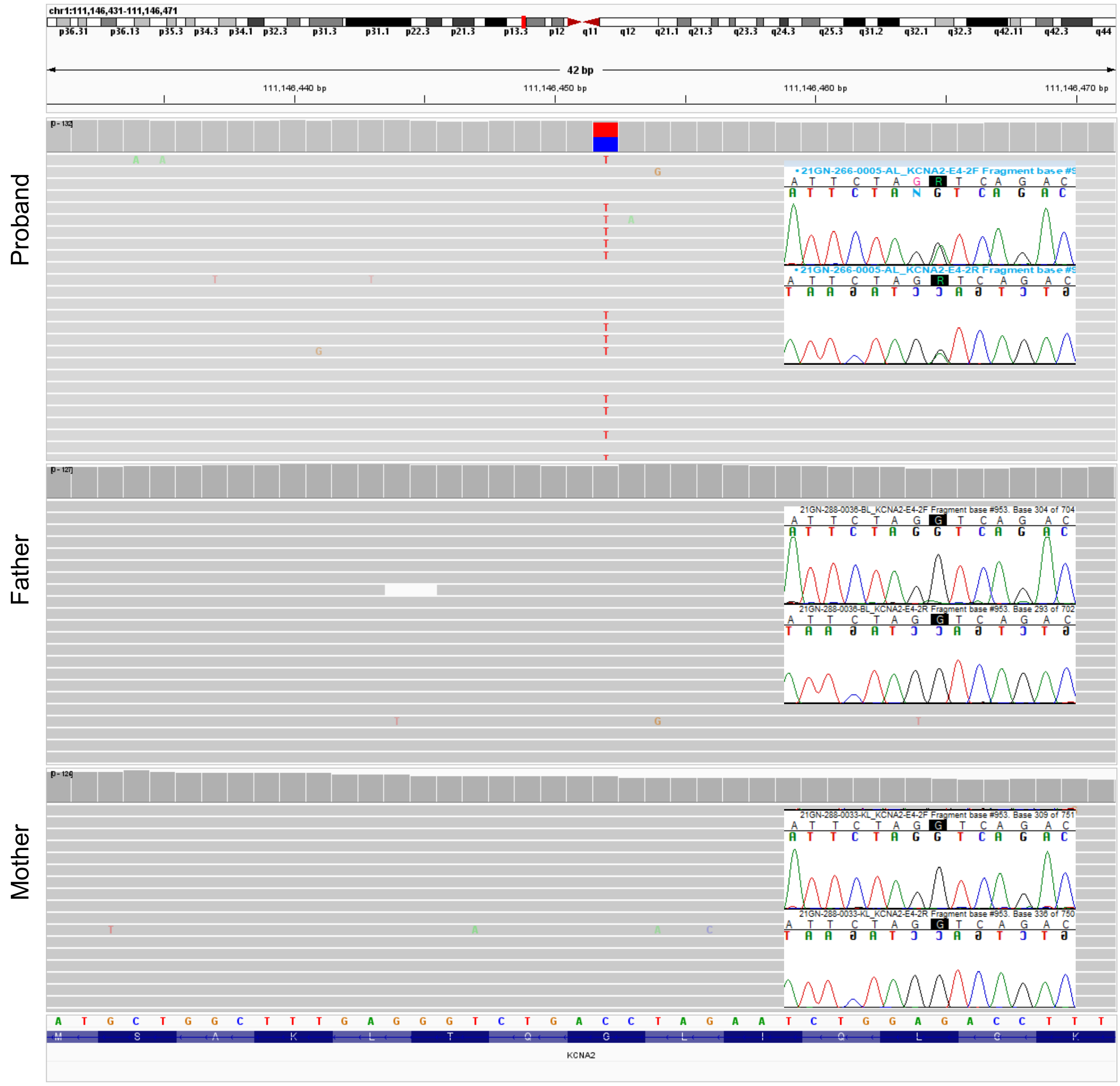

Figure S1: Integrated genomics viewer (IGV) visualization of the *KCNA2* variant in trio sequencing data. The proband, shows a heterozygous C>T substitution at chr1:111,146,452 (GRCh37), corresponding to NM_004974.4:c.953G>A (p.Gly318Asp). The alternate T allele (red) is supported by 60 of 121 sequencing reads (50%) with balanced forward and reverse strand representation (30+, 30−), while 58 reads (48%) support the reference C allele (29+, 29−). Low-level non-reference bases (A: 1%, G: 2%) are consistent with background sequencing noise. Individual sequencing reads are shown as grey bars with mismatched bases highlighted in color (T in red), and coverage histograms above each track indicate read depth and allele proportions. Both parents (mother, father) show only the reference allele at this position with no evidence of the alternate allele, consistent with a *de novo* variant in the proband. Insets show Sanger-sequencing chromatograms.
